# Oncogenic functions of hMDMX in *in vitro *transformation of primary human fibroblasts and embryonic retinoblasts

**DOI:** 10.1186/1476-4598-10-111

**Published:** 2011-09-12

**Authors:** Kristiaan Lenos, Job de Lange, Amina FAS Teunisse, Kirsten Lodder, Matty Verlaan-de Vries, Eliza Wiercinska, Marja JM van der Burg, Karoly Szuhai, Aart G Jochemsen

**Affiliations:** 1 Department of Molecular Cell Biology, Leiden University Medical Center, P.O. Box 9600, 2300 RC Leiden, The Netherlands

**Keywords:** Transformation model, p53 pathway, tumorigenesis, hMDMX, hMDM2, retinoblastoma, Nutlin-3

## Abstract

**Background:**

In around 50% of all human cancers the tumor suppressor p53 is mutated. It is generally assumed that in the remaining tumors the wild-type p53 protein is functionally impaired. The two main inhibitors of p53, hMDM2 (MDM2) and hMDMX (MDMX/MDM4) are frequently overexpressed in wild-type p53 tumors. Whereas the main activity of hMDM2 is to degrade p53 protein, its close homolog hMDMX does not degrade p53, but it represses its transcriptional activity. Here we study the role of hMDMX in the neoplastic transformation of human fibroblasts and embryonic retinoblasts, since a high number of retinoblastomas contain elevated hMDMX levels.

**Methods:**

We made use of an *in vitro *transformation model using a retroviral system of RNA interference and gene overexpression in primary human fibroblasts and embryonic retinoblasts. Consecutive knockdown of RB and p53, overexpression of SV40-small t, oncogenic HRasV12 and HA-hMDMX resulted in a number of stable cell lines representing different stages of the transformation process, enabling a comparison between loss of p53 and hMDMX overexpression. The cell lines were tested in various assays to assess their oncogenic potential.

**Results:**

Both p53-knockdown and hMDMX overexpression accelerated proliferation and prevented growth suppression induced by introduction of oncogenic Ras, which was required for anchorage-independent growth and the ability to form tumors *in vivo*. Furthermore, we found that hMDMX overexpression represses basal p53 activity to some extent. Transformed fibroblasts with very high levels of hMDMX became largely resistant to the p53 reactivating drug Nutlin-3. The Nutlin-3 response of hMDMX transformed retinoblasts was intact and resembled that of retinoblastoma cell lines.

**Conclusions:**

Our studies show that hMDMX has the essential properties of an oncogene. Its constitutive expression contributes to the oncogenic phenotype of transformed human cells. Its main function appears to be p53 inactivation. Therefore, developing new drugs targeting hMDMX is a valid approach to obtain new treatments for a subset of human tumors expressing wild-type p53.

## Background

In approximately 50% of all human cancers mutations are found in the *TP53 *gene, encoding the tumor suppressor protein p53 [1,2], whereas it is assumed that in tumors expressing wild-type p53 the tumor suppressing activity of p53 is attenuated [3]. Normal, non-stressed cells maintain relatively low p53 protein levels. Upon various stress signals like DNA damage or oncogenic stress, p53 is stabilized and activated. Activated p53 affects various processes, including cell cycle progression, DNA repair, senescence and apoptosis [4]. Two main negative regulators of p53 are MDM2 and MDMX, also called hMDM2 and hMDMX. MDM2, an E3 ubiquitin ligase, inhibits p53 via poly-ubiquitination [5] and by binding to p53's N-terminus, thereby shielding its transcription activation domain. Since the *MDM2 *gene is also a p53 target, a negative feedback-loop is established [6]. The importance of MDM2 in p53 regulation was best shown by the p53-dependent embryonic lethality of *MDM2 -/- *mice [7,8]. Similarly, *MDMX *-/- mice are embryonic lethal in a p53-dependent manner [9-11], indicating that both MDM2 and MDMX fulfil an essential, non-redundant function in p53-regulation. Despite great structural similarities between MDM2 and MDMX [12], including the RING finger domain needed for MDM2 E3 ligase activity, MDMX has no detectable E3 ligase activity. MDMX functions mostly by inhibiting p53 activity through interaction with its transcription activation domain [13,14]. Furthermore, MDMX and MDM2 dimerize via their RING finger domains [15], thereby stabilizing MDM2 and promoting its E3 ligase activity towards p53 [16,17].

hMDM2 is overexpressed in 5-10% of all human tumors, revealing hMDM2 as an oncogene [18]. Similar observations were made regarding hMDMX. A study of common tumor types showed increased *hMDMX *mRNA expression in 20% of these tumors [19], and a subset of gliomas contained *hMDMX *gene amplification [20]. Furthermore, Ramos *et al*. showed upregulated or aberrant hMDMX expression in a large number of human tumor cell lines, mostly correlating with wild-type p53 status [21]. A particularly high proportion of retinoblastomas contain *hMDMX *gene amplification [22]. hMDMX knockdown in p53 wild-type tumor cells has been shown to induce p53-dependent growth inhibition [19,22].

The first evidence for direct oncogenic activity of MDMX was provided by Danovi *et al*. [19]. MDMX overexpression in early cultures of mouse embryonic fibroblasts resulted in immortalization and neoplastic transformation when combined with HRasV12 overexpression. This suggests that MDMX overexpression is sufficient to inactivate the p53 tumor suppressor pathway. However, such an oncogene function of hMDMX has not yet been directly shown in human cells.

Human primary cells require a specific set of genetic changes for neoplastic transformation. By expression of the human Telomerase reverse transcriptase subunit (hTERT), oncogenic HRasV12, and the early region of SV40, encoding the viral large and small T antigens (LT and st), primary human cells can be immortalized and transformed. LT is needed to inactivate RB and p53, since functional loss of both genes is required for tumor formation [23,24]. By combining this transformation model with specific RNA interference, the tumor-suppressive functions of *p14^ARF ^*and *p16^INK4A ^*were assessed by Voorhoeve and Agami [25]. They also showed that directly targeting *p53 *and *RB *could replace LT expression. Here we use a retroviral system of RNA interference and gene overexpression to establish an *in vitro *transformation model for assessing the contribution of hMDMX to the transformation of human primary cells.

## Results

### Generation of transformed human skin fibroblasts and human embryonic retinoblasts, including hMDMX as a potential oncogene

To investigate whether *hMDMX *can function as an oncogene in the transformation of human primary cells, we applied a previously described *in vitr*o transformation model [25] to two different cell types: human foreskin fibroblasts (VH10) and human embryonic retinoblasts (HER).

**Figure 1:**
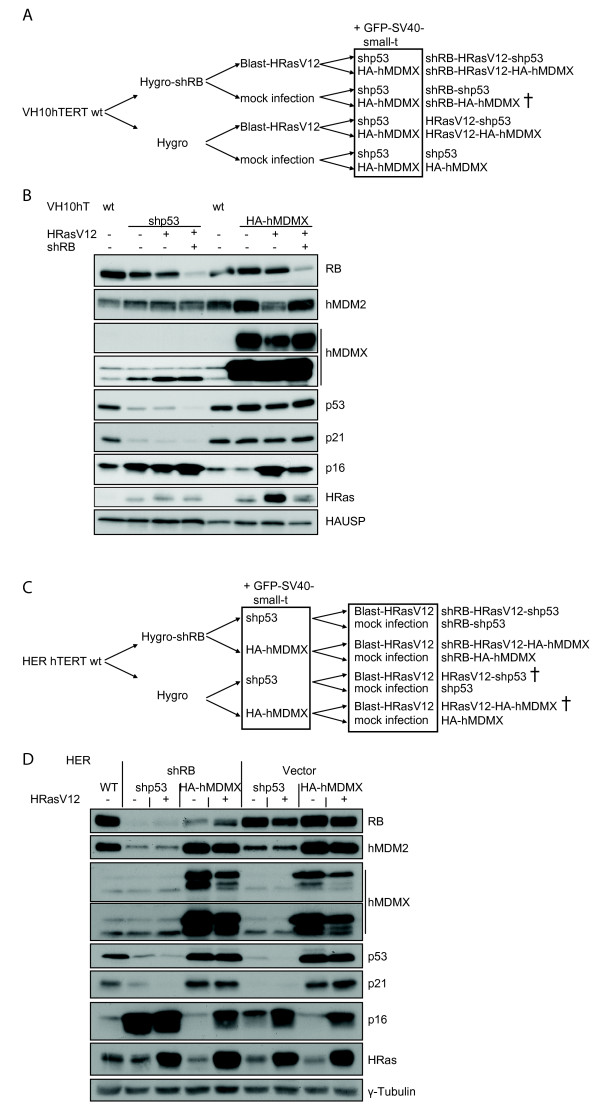
**Generation of panels of transformed human skin fibroblasts and human embryonic retinoblasts**. A. Schematic representation of transformation process. Primary human fibroblasts (VH10) were immortalized with human Telomerase (hTERT). In subsequent rounds of retroviral infection using the indicated constructs, followed by selection, several stable cell lines were created. B. Total cell extracts from all transformed fibroblast cell lines were analyzed by immunoblotting with the indicated antibodies. C. Human Embryonic Retinoblasts (HER) were similarly transformed according to the scheme. D. Total cell extracts from all transformed HER cell lines were analyzed by immunoblotting with the indicated antibodies.

The generation of the VH10 transformation model is depicted in Figure 1A. Sequential retroviral transductions resulted in a panel of stable polyclonal cell lines, representing different stages of the transformation process. This enabled a pair-wise comparison between hMDMX overexpression and p53-knockdown. Stable cell lines with shRB-HA-hMDMX, or shRB alone, could not be established, suggesting that RB reduction is growth limiting in these cells. Oncogenic HRasV12 without concomitant p53-knockdown or HA-hMDMX overexpression induced a senescent-like crisis blocking proliferation of most cells, followed by expansion of single colonies. This suggests the occurrence of additional selection bypassing the HRasV12-induced senescence. RB knockdown was not sufficient to rescue HRasV12-induced growth inhibition. Therefore, the cell lines VH10-shRB-HRasV12 and VH10-HRasV12 could not be established. We monitored the effectiveness of the transductions by western blotting (Figure 1B) and found strong overexpression of hMDMX, somewhat increased HRas levels and marked reductions of p53 and RB, correlating with the respective transductions. Interestingly, endogenous HRas level was slightly increased upon p53-knockdown or hMDMX overexpression, correlating with accelerated growth. HRasV12 has been reported to induce p16-dependent senescence in human fibroblasts [4,25,26]. Indeed, HRasV12-transformed as well as p53-knockdown cells expressed higher p16 protein levels (Figure 1B), although we observed no signs of senescence. This suggests that during the transformation process the pathway downstream of p16 is somehow impaired. Alternatively, p53 inactivation may prevent HRasV12-induced senescence, which was indeed described for hMDMX overexpression [27].

**Figure 2:**
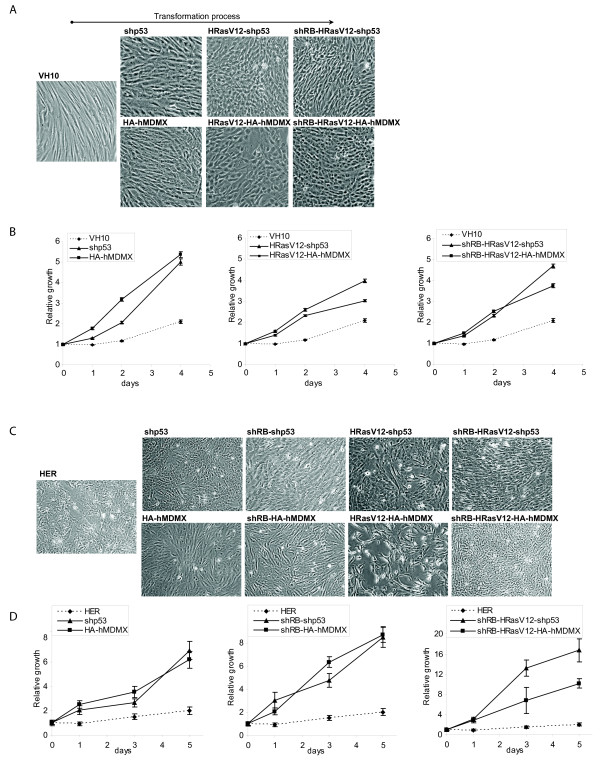
**Transformation alters cell morphology and growth rate**. Phase-contrast photographs of the transformed VH10 (A) and HER (C) cell lines (10 × magnification, Olympus CKX41) showing morphology changes during the transformation process. Growth rates of VH10 (B) and HER (D) cell lines were measured using WST-1 proliferation assays.

For HER transformation, we initially used a comparable approach. However, HRasV12-transformed cells could not be established without p53-knockdown or HA-hMDMX overexpression, which prompted us to modify the scheme (Figure 1C). Transformation of HER-hTERT-shRB or HER-hTERT cells with an empty puromycin vector and subsequent puromycin selection resulted in initial colony formation, but these colonies eventually stopped growing. This suggests that in HER cells, in contrast to VH10, inactivation of p53 is essential for establishing immortalized cell lines. Protein levels of RB, hMDMX, p53 and HRas correlated with the applied transductions (Figure 1D), although the RB depletion was less efficient in HA-hMDMX cells (lane 4 and 5). Similar to the observations in VH10 cells, HRasV12 expression induced p16 protein levels in HER cells. This did not affect growth of RB-knockdown cells, whereas HER cells without RB-knockdown eventually stopped proliferating upon HRasV12 overexpression. Likely, HRasV12 activated a p16- and RB-dependent mechanism resulting in growth suppression (also illustrated by large, flattened cells, Figure 2C), which could not be rescued by p53-knockdown or hMDMX overexpression. Therefore, these cells could not be used in further experiments.

hMDMX overexpression and p53-knockdown were analyzed with qRT-PCR, showing approximately 90% reduction of *p53 *and 10-fold increase of *hMDMX *mRNA expression in VH10 cells (Table 1 and not shown). Expression of p53 target genes *p21, PUMA *and *hMDM2-p2 *was significantly decreased upon p53-knockdown in VH10 and HER cells, and also hMDMX overexpression slightly decreased basal levels of some of these genes (Table 1). Reduced p21 protein levels upon p53-knockdown (Figure 1B and 1D), in line with the qRT-PCR data, indicate impaired basal p53 activity. hMDMX overexpression did slightly reduce basal p21 protein levels in VH10 cells (Table 2), In addition, in HA-hMDMX cells the protein levels of p53 were slightly increased, most likely by protein stabilization.

**Table 1:** mRNA expression levels in VH10 and HER cell lines

VH10	*p21*			*hMDM2-p2*			*PUMA*		
**VH10 wt**	1.00	±	0.16	1.00	±	0.17	1.00	±	0.23
**shp53**	0.24	±	0.03	0.20	±	0.03	0.49	±	0.11
**HRasV12-shp53**	0.15	±	0.03	0.32	±	0.07	0.44	±	0.15
**shRB-HRasV12-shp53**	0.63	±	0.11	0.41	±	0.15	1.54	±	0.32
**HA-hMDMX**	0.47	±	0.09	0.61	±	0.08	0.41	±	0.08
**HRasV12-HA-hMDMX**	0.76	±	0.12	1.05	±	0.17	0.53	±	0.14
**shRB-HRasV12-HA-hMDMX**	1.05	±	0.12	1.14	±	0.15	0.28	±	0.07

**HER**	***p21***			***hMDM2-p2***			***PUMA***		

**HER wt**	1.00	±	0.16	1.00	±	0.15	1.00	±	0.19
**shp53**	0.11	±	0.03	0.01	±	0.01	0.46	±	0.13
**shRB-shp53**	0.23	±	0.05	0.02	±	0.00	0.36	±	0.06
**shRB-HRasV12-shp53**	0.05	±	0.02	0.01	±	0.00	0.15	±	0.04
**HA-hMDMX**	1.51	±	0.26	1.43	±	0.28	0.67	±	0.30
**shRB-HA-hMDMX**	1.01	±	0.16	0.62	±	0.13	0.34	±	0.11
**shRB-HRasV12-HA-hMDMX**	2.52	±	0.70	0.61	±	0.17	0.60	±	0.17

Total RNA of each cell line was isolated and expression levels of the indicated genes were determined by qRT-PCR and normalized for the housekeeping genes *CAPNS1 *and *TBP*; levels are shown relative to wild-type cells.

**Table 2:** Protein levels, relative to untreated VH10 wt cells, corrected for HAUSP expression

Cell line	Nutlin-3 treatment (h)	p53	p21	hMDM2
	**0**	1.00	1.00	1.00
**VH10 wt**	**6**	4.96	6.68	82.32
	**24**	7.82	11.16	149.60

	**0**	0.35	0.20	0.27
**shp53**	**6**	0.87	0.65	0.30
	**24**	1.25	2.26	4.54

	**0**	0.28	0.03	0.33
**HRasV12-shp53**	**6**	0.30	0.09	0.78
	**24**	0.44	0.33	0.74

	**0**	0.45	1.01	2.51
**shRB-HRasV12- shp53**	**6**	0.49	1.24	4.55
	**24**	0.74	2.85	9.17

	**0**	6.99	0.56	0.87
**HA-hMDMX**	**6**	9.72	2.86	47.81
	**24**	11.70	2.10	17.19

	**0**	1.63	0.63	0.69
**HRasV12-HA-hMDMX**	**6**	4.13	2.88	36.75
	**24**	5.01	3.07	29.42

	**0**	1.07	1.45	2.79
**shRB-HRasV12-HA-hMDMX**	**6**	2.38	5.37	132.80
	**24**	1.76	4.28	79.91

**Figure 3:**
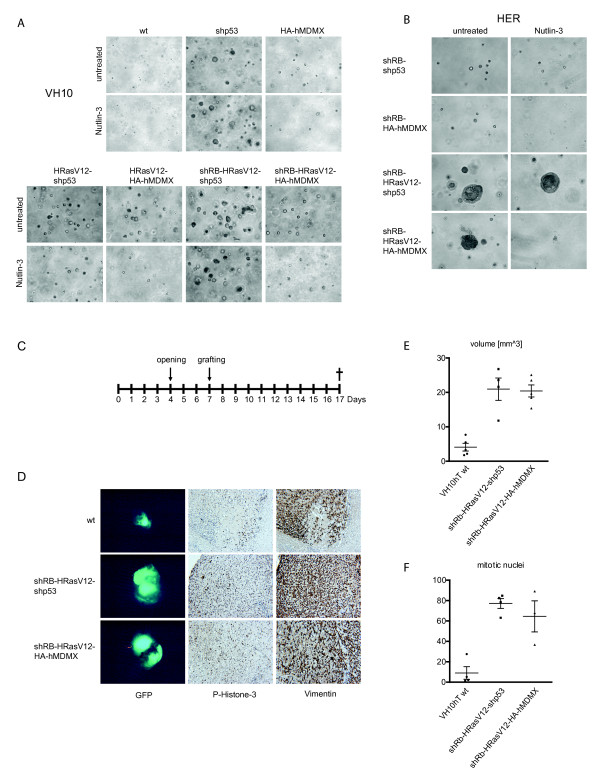
**hMDMX overexpression promotes anchorage-independent growth and tumor growth *in vivo***. A, B. Various VH10hTERT and HER cell lines were embedded in 0.3% agarose on a 0.6% agarose bottom-layer, with additional normal growth medium or growth medium containing 10 μM Nutlin-3 on top of the agarose. Colony outgrowth was monitored and pictures were taken 18 days (VH10) and 4 weeks (HER) after seeding. Representative pictures of several independent experiments are shown. C. Schedule for investigating *in vivo *growth of parental and transformed VH10 cells using the shell-less chicken CAM model. At embryonic development day (EDD) 7, 2.5 million cells were grafted onto a chicken CAM. Tumors were harvested at EDD17. D. Representative pictures of GFP-positive tumors, P-Histone-3 staining and vimentin staining. E. Tumor volumes of VH10hTERT control (N = 5), shRB-HRasV12-shp53 (N = 4) and shRB-HRasV12-HA-hMDMX (N = 5). F. Quantification of the number of mitotic (P-Histone-3 positive) cells. Statistical analysis was performed by one-way ANOVA followed by Bonferroni's Multiple Comparison Test. P < 0.05 was considered statistically significant.

Band intensities shown in figure 3B were quantified using the Odyssey 2.1 analysis software (LI-COR Biosciences) and the relative protein levels were calculated using HAUSP expression as an internal control. Basal protein levels in VH10 wild-type cells were set at 1.0.

Immunofluorescence analysis revealed abundant GFP throughout the entire cell in all established HER and VH10 cell lines, which confirmed SV40-st expression (Additional File 1 Figure S1A and S1C).

Exogenous hMDMX showed mainly cytoplasmic localisation in VH10 cells, but nuclear hMDMX was also observed (Additional File 1 Figure S1A). In HER cells, the main localization was nuclear (Additional File 1 Figure S1B). High levels of cytoplasmic hMDMX were reported to prevent p53 nuclear localization [28]. However, we found no alterations in p53 localization upon hMDMX overexpression (Additional File 1 Figure S1B and S1D). hMDM2 protein, irrespective of hMDMX levels, was detected in the nucleus, although it could be observed in the cytoplasm as well. The cytoplasmic signal of hMDM2 is relatively underrepresented since the protein is diffused throughout the relatively large cytoplasmic surface, but is certainly present, as reported before [29] (Additional File 1 Figure S1B and S1D).

Since p53 contributes to the maintenance of genomic stability [4], the various cell lines were analyzed for chromosomal abnormalities using COBRA-FISH [30]. Wild-type VH10hTERT cells (Additional File 2 Figure S2A) showed a normal 46, XY karyogram in 20% of the analyzed cells. The remaining cells harboured a Robertsonian translocation [31], which results in loss of the short arms of two acrocentric chromosomes. As these contain the ribosomal gene cassettes, this translocation has no further consequences at the cellular level. The observed rob(13;22) chromosome was lost during the transformation process. The transformed VH10 cell lines showed heterogeneous populations of mainly diploid and chromosomal stable cells, with low percentage random translocations or polyploidy. The HER cells showed more chromosomal aberrations and translocations (Additional File 2 Figure S2B). Most notably, loss of chromosome 13, harbouring the RB gene, was found in four out of six cell lines. The fact that it was not found in the HA-hMDMX and shRB-HA-hMDMX-HRasV12 cell line suggests that loss of this chromosome occurred independently in those four cell lines (see Figure 1C for transformation scheme). Two unlinked cell lines (shp53 and shRB-HA-hMDMX-HRasV12) lost one X-chromosome, whereas loss of chromosome 22 and gain of chromosome 8 in shRB-shp53 and shRB-shp53-HRasV12 is likely to have been passed on from their shared parental cell line. In addition, several random translocations and fusions were observed, however, none was found in more than one cell line. In conclusion, transformation of VH10 and HER cells did not induce wide-spread genomic instability and aneuploidy.

### Alterations in morphology and proliferation rate during transformation process

Cell morphology changed during the transformation process. Whereas normal VH10hTERT cells are extended, fibroblastic cells aligning orderly in the dish, during the sequential transformation stages the cells became apparently smaller and rounder (Figure 2A). HRasV12 induced a disordered way of growing and showed loss of contact inhibition. hMDMX-overexpressing and p53-knockdown cells showed similar morphology changes. Both shp53- and HA-hMDMX cells obviously proliferated faster than wild-type VH10hTERT cells, a property that was not further enhanced by additional HRasV12 expression. This was confirmed in short-term growth (WST-1) assays (Figure 2B), suggesting that hMDMX overexpression is sufficient to inhibit p53-dependent growth control, similar to p53-knockdown.

Morphology changes in transformed HER cells were comparable to those observed in VH10 cells (Figure 2C). In addition, both p53-knockdown and hMDMX overexpression accelerated growth as compared to wild-type HER cells (Figure 2D). HRasV12 even further enhanced growth rate in shRB-shp53 cells, but not in shRB-HA-hMDMX cells.

### Effect of hMDMX overexpression on anchorage-independent growth

Anchorage-independent growth is a vital feature of tumorigenic cells. Therefore, we investigated the growth potential of the different cell lines in soft agar (Figure 3A).

Control VH10hTERT cells did not grow, but both p53-knockdown and hMDMX overexpression induced formation of some small colonies. HRasV12 expression clearly increased the size and the number of colonies. However, this was more pronounced in p53-knockdown cells than in hMDMX-overexpressing cells. Interestingly, hMDMX overexpression did not prevent the growth inhibitory effect of Nutlin-3, in contrast to experiments in 2D-culture (see below). These findings suggest that hMDMX cannot fully inhibit the function of p53 in soft agar growth.

Untransformed HER cells did not grow at all in soft agar, and neither hMDMX overexpression nor p53-knockdown alone was able to induce colony formation (not shown). Although additional RB knockdown induced formation of very small colonies, only HRasV12 expression dramatically increased colony size and number (Figure 3B). Similar to the observations in VH10 cells, colony formation was more efficient in p53-knockdown cells than in hMDMX-overexpressing cells. Furthermore, Nutlin-3 inhibited colony formation of hMDMX-overexpressing cells, whereas p53-knockdown cells were unaffected.

### Role of hMDMX in tumorigenicity *in vivo*

We tested the *in vivo *tumorigenic potential of the transformed VH10 cells by subcutaneous injection into Balb/c nu/nu mice. Unfortunately, no tumor formation could be detected. The lack of tumor growth could possibly be explained by the immune response still present in nu/nu mice; however, similar results were obtained in NOD/SCID mice. Therefore, we switched to the shell-less chick CAM assay [32] (Figure 3C-F). Ten days after grafting both shRB-HRasV12-HA-hMDMX- and shRB-HRasV12-shp53 tumors were significantly larger (4-5 fold) than those formed by wild-type VH10 cells (P < 0.05, Bonferroni's Multiple Comparison Test; Figure 3D left and E). Interestingly, the transformed cell lines showed equal tumor volumes, indicating that hMDMX overexpression and p53-knockdown have similar effects on tumor growth. Moreover, tumors from the transformed cells contained significantly (P < 0.05) more mitotic cells than wild-type tumors as revealed with P-Histone-3 staining, with no detectable difference between hMDMX-overexpressing and p53-knockdown cells (Figure 3D middle and 3F). Staining with fibroblast marker vimentin showed the fibroblastic origin of the tumors (Figure 3D right).

The *in vivo *growth capacity of transformed HER cells was tested in a previously described murine model [33], by injection into the anterior eye chamber of Balb/c nu/nu mice. Both tested cell lines showed similar, but limited *in vivo *growth potential. The hMDMX-expressing cells showed growth in 2/5 cases, but growth stopped when the eye chamber was filled up to 50% with tumor cells. The p53-knockdown cells started tumor growth in 4/5 cases, but stopped when the eye chamber was filled up to 20% (2x), 30% (1x) or 50% (1x). Altogether, it is clear that hMDMX overexpression promotes *in vivo *tumor growth and in that respect largely mimics p53-knockdown in the same cells.

### hMDMX overexpression inhibits the Nutlin-3 induced p53 response in VH10 skin fibroblasts

**Figure 4:**
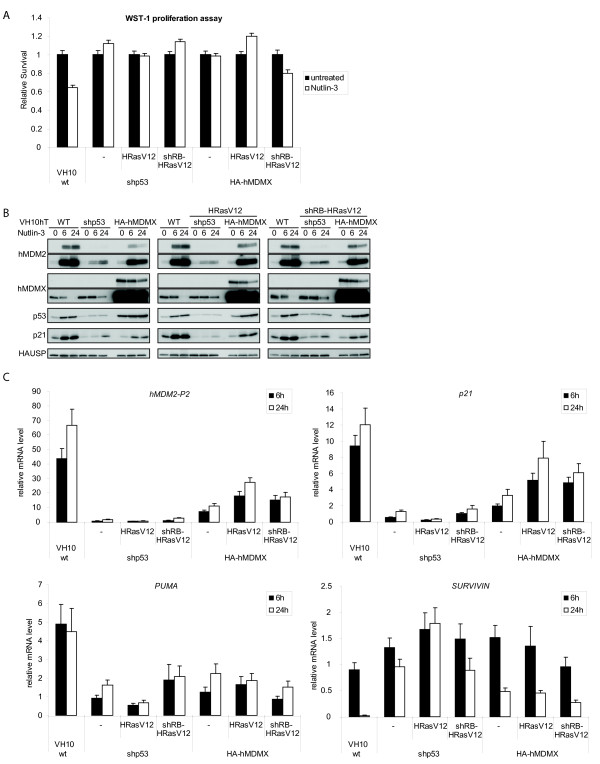
**hMDMX overexpression inhibits Nutlin-3 mediated p53 activation in human fibroblasts**. A. Various VH10hTERT cell lines were continuously treated with 10 μM Nutlin-3 and proliferation was measured after 96 hours using a WST-1 assay. Relative cell numbers are displayed as survival relative to untreated cells. B. Various VH10hTERT cell lines were treated for the indicated times with 10 μM Nutlin-3, and protein levels were analyzed with immunoblotting using the indicated antibodies. C. qRT-PCR analysis of cells treated as in B. Expression levels of *hMDM2-p2, p21, PUMA *and *SURVIVIN *are shown as the fold induction relative to untreated wild-type VH10 cells.

We next investigated whether hMDMX overexpression prevents p53 activation in VH10 cells. The various cell lines were treated with the small-molecule p53-activator Nutlin-3 [34]. Nutlin-3 reduced survival of normal human fibroblasts (Figure 4A), whereas p53-knockdown cells were not affected. hMDMX overexpression also prevented Nutlin-3 induced growth inhibition. In wild-type VH10hTERT cells, the Nutlin-3 response is marked by increased p53, hMDM2 and p21 and decreased hMDMX protein levels (Figure 4B). This response was diminished upon p53-knockdown, reaching levels slightly above basal expression in wild-type cells (Table 2). HA-hMDMX overexpression also attenuated the induction of p53 and its target genes. Notably, exogenous HA-hMDMX levels remained relatively high, despite some Nutlin-3 induced degradation. Levels of the p53-responsive transcripts of *hMDM2-p2, p21 *and *PUMA *correlated with protein levels (Figure 4C). Furthermore, hMDMX overexpression partially rescued the reduction of the anti-apoptotic gene *SURVIVIN *by Nutlin-3 [35]. Table 3 shows fold changes of *p53, hMDM2, hMDMX *and the p53 targets *hMDM2-p2, p21, PUMA, GADD45-alpha *and *SURVIVIN*, for each cell line separately. As expected, *p53 *and *hMDMX *mRNA levels did not significantly change upon Nutlin-3 treatment.

**Table 3:** Fold induction mRNA per cell line after Nutlin-3 treatment

Cell line	Nutlin-3 treatm (h)	*p53*			*p21*			*hMDM2*			*hMDM2-p2*			*hMDMX *			*PUMA*			*GADD45a*			*SURVIVIN*		
**VH10 wt**	**0**	1.00	±	0.19	1.00	±	0.16	1.00	±	0.16	1.00	±	0.17	1.00	±	0.16	1.00	±	0.23	1.00	±	0.38	1.00	±	0.16
	**6**	1.10	±	0.18	9.36	±	1.34	28.62	±	5.40	43.93	±	6.93	1.38	±	0.21	4.90	±	1.06	5.17	±	1.45	0.90	±	0.14
	**24**	0.79	±	0.18	11.98	±	2.09	26.42	±	5.42	66.60	±	11.19	0.95	±	0.16	4.49	±	1.25	4.89	±	1.79	0.02	±	0.00

**shp53**	**0**	1.00	±	0.09	1.00	±	0.11	1.00	±	0.25	1.00	±	0.09	1.00	±	0.09	1.00	±	0.23	1.00	±	0.11	1.00	±	0.13
	**6**	0.85	±	0.17	2.24	±	0.24	1.56	±	0.31	4.26	±	0.43	0.91	±	0.17	1.84	±	0.35	1.46	±	0.28	1.18	±	0.14
	**24**	0.63	±	0.54	5.36	±	0.45	4.08	±	0.92	9.46	±	0.73	1.00	±	0.12	3.29	±	0.54	1.28	±	0.12	0.85	±	0.11

**HRasV12-shp53**	**0**	1.00	±	0.35	1.00	±	0.27	1.00	±	0.32	1.00	±	0.28	1.00	±	0.29	1.00	±	0.41	1.00	±	0.28	1.00	±	0.23
	**6**	0.81	±	0.24	1.34	±	0.33	1.29	±	0.39	1.97	±	0.56	1.09	±	0.41	1.19	±	0.40	1.14	±	0.30	0.99	±	0.22
	**24**	0.84	±	0.28	2.11	±	0.50	1.44	±	0.36	2.37	±	0.66	1.18	±	0.30	1.53	±	0.47	1.37	±	0.32	1.06	±	0.22

**shRB-HRasV12-shp53**	**0**	1.00	±	0.24	1.00	±	0.18	1.00	±	0.22	1.00	±	0.48	1.00	±	0.19	1.00	±	0.18	1.00	±	0.18	1.00	±	0.18
	**6**	0.88	±	0.24	1.54	±	0.30	1.16	±	0.30	2.61	±	0.97	1.22	±	0.24	1.22	±	0.54	1.49	±	0.63	1.00	±	0.20
	**24**	0.98	±	0.27	2.49	±	0.67	1.68	±	0.66	6.32	±	2.59	1.18	±	0.38	1.35	±	0.35	1.76	±	0.48	0.59	±	0.16

**HA-hMDMX**	**0**	1.00	±	0.17	1.00	±	0.22	1.00	±	0.03	1.00	±	0.08	1.00	±	0.34	1.00	±	0.14	1.00	±	0.09	1.00	±	0.58
	**6**	0.74	±	0.23	4.14	±	0.67	3.47	±	1.26	11.83	±	1.00	1.42	±	0.36	3.05	±	0.49	1.25	±	0.58	1.52	±	0.64
	**24**	1.63	±	0.25	6.86	±	1.71	11.92	±	1.36	18.14	±	2.03	1.80	±	0.58	5.45	±	1.00	2.88	±	0.93	0.48	±	0.20

**HRasV12-HA-hMDMX**	**0**	1.00	±	0.14	1.00	±	0.16	1.00	±	0.16	1.00	±	0.16	1.00	±	0.22	1.00	±	0.29	1.00	±	0.14	1.00	±	0.16
	**6**	0.90	±	0.16	6.78	±	1.16	9.01	±	1.83	17.33	±	2.97	1.09	±	0.22	3.09	±	0.91	1.27	±	0.21	0.79	±	0.22
	**24**	0.79	±	0.10	10.38	±	2.76	12.08	±	1.58	26.21	±	2.95	0.74	±	0.11	3.50	±	0.85	1.69	±	0.19	0.26	±	0.03

**shRb-HRasV12-HA-hMDMX**	**0**	1.00	±	0.10	1.00	±	0.05	1.00	±	0.09	1.00	±	0.07	1.00	±	0.05	1.00	±	0.26	1.00	±	0.05	1.00	±	0.06
	**6**	1.04	±	0.13	4.57	±	0.46	6.46	±	1.61	13.53	±	2.08	1.01	±	0.13	3.10	±	0.63	1.73	±	0.26	0.81	±	0.13
	**24**	0.99	±	0.14	5.76	±	0.92	6.55	±	0.94	15.36	±	2.04	0.96	±	0.14	5.33	±	1.27	1.71	±	0.28	0.23	±	0.03

VH10 cells were treated with 10 μM Nutlin-3 for the indicated times and analyzed using qRT-PCR. Expression was normalized for the housekeeping genes *CAPNS1 *and *TBP*. The induction of mRNA expression upon Nutlin-3 treatment per cell line is shown for *p53, p21, hMDM2, hMDM2-p2, hMDMX, PUMA, GADD45alpha *and *SURVIVIN*. For each cell line the basal mRNA expression is set at 1.0.

### hMDMX overexpression in HER cells is not sufficient to inhibit the Nutlin-3 induced p53 response

**Figure 5:**
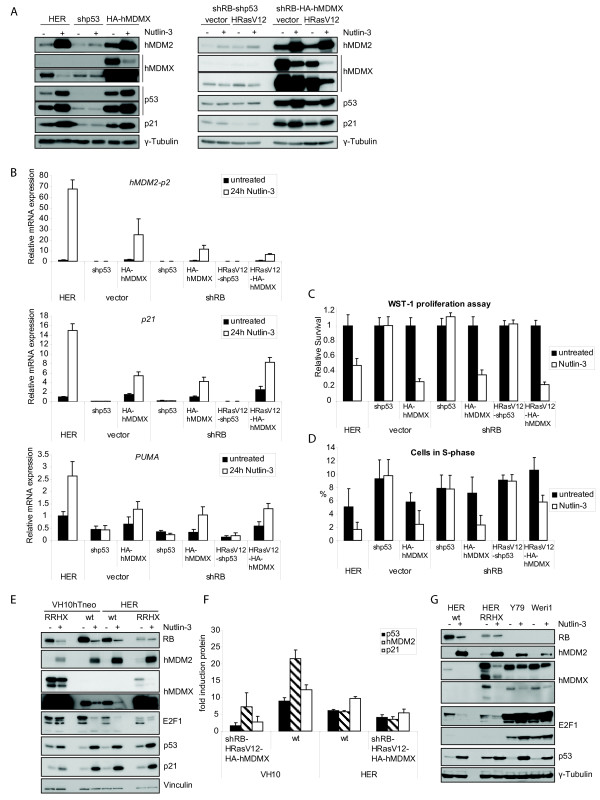
**hMDMX overexpression in HER cells is not sufficient to prevent the Nutlin-3 induced p53-activation, which resembles retinoblastoma cell lines**. A. The various HER cell lines were treated with 10 μM Nutlin-3 for 24 hours, and protein levels were analyzed with immunoblotting using the indicated antibodies. B. qRT-PCR analysis of cells treated as in A. Expression levels of *hMDM2-p2, p21 *and *PUMA *are shown as the fold induction relative to untreated wild-type HER cells. C. The various HER cell lines were continuously treated with 10 μM Nutlin-3 and proliferation was measured after 120 hours using a WST-1 assay. Relative cell numbers are displayed as survival relative to untreated cells. D. Cells were treated with 10 μM Nutlin-3 for 24 hours and analyzed by flow cytometry. Percentages of cells in S-phase are displayed as indicative for cell proliferation. E. Parental and shRB-HRasV12-HA-hMDMX (RRHX) transformed VH10 and HER cells were treated with 10 μM Nutlin-3 for 24 hours and analyzed with immunoblotting using the indicated antibodies. F. Quantification of the indicated protein levels using Odyssey 2.1 analysis software (LI-COR Biosciences) for at least two different exposures. Relative protein levels were calculated using Vinculin expression as an internal control and indicated as fold induction relative to untreated cells. G. Parental and RRHX transformed HER cells and the retinoblastoma cell lines Y79 and Weri1 were treated with 10 μM Nutlin-3 for 24 hours, and analyzed with immunoblotting using the indicated antibodies.

Similar to the observations in VH10, in HER cells Nutlin-3 increased p53, hMDM2 and p21 and reduced hMDMX protein levels, which was efficiently blocked by p53-knockdown (Figure 5A). Surprisingly however, hMDMX overexpression hardly rescued these effects. Although at the mRNA level (Figure 5B) the inductions of p53 targets *hMDM2-p2, p21 *and *PUMA *were indeed slightly attenuated, this appeared to be insufficient to prevent Nutlin-3 induced growth inhibition, as illustrated by reduced survival (Figure 5C) and S-phase depletion (Figure 5D).

To explain the differences between the hMDMX-overexpressing VH10 and HER cells in their Nutlin-3 response, we compared protein and mRNA levels side-by-side (Figure 5E and 5F). Strikingly, HA-hMDMX was clearly higher expressed in VH10 cells than in HER cells, while endogenous p53, hMDM2 and p21 levels were comparable. As Nutlin-3 not only binds hMDM2 but also hMDMX, albeit with much lower affinity [22], the levels of hMDMX may affect Nutlin-3 sensitivity. In HER cells, the remaining hMDMX levels after Nutlin-3 treatment may not be sufficient to prevent p53 activity. Importantly, we found that hMDMX levels in transformed HER cells were comparable to the levels in retinoblastoma cell lines Y79 and Weri1 (Figure 5G). We have previously shown that in these retinoblastoma cells p53 is inhibited via high hMDMX expression, and that they are sensitive to Nutlin-3 [22]. These findings indicate that the transformed retinoblasts provide a representative model for retinoblastoma.

The response to Nutlin-3 may also be determined by E2F1 activity, which activates p73. Kitagawa *et al*. [36] reported that Nutlin-3-induced downregulation of E2F1 correlates with relative Nutlin-3 resistance, and they suggested that cells lacking RB activity are much more prone to entering Nutlin-3-induced apoptosis. Therefore, we compared RB and E2F1 levels in a selected panel of VH10 and HER cell lines (Figure 5E). As reported before [37], Nutlin-3 decreased total and hyper-phosphorylated (upper band) RB. However, the amount of hypo-RB (lower band) was hardly affected. Moreover, after Nutlin-3 treatment, the hypo-RB levels in normal and RB-knockdown cells were comparable, and the differences between VH10 and HER cells were rather small. E2F1 reduction at the protein (Figure 5E) and mRNA level (Additional File 3 Figure S3A, upper panel) as observed in the parental VH10 and HER cells was attenuated in the transformed cells. The mRNA levels of the E2F1 target gene *CDC25a *followed a similar pattern. Nutlin-3 also reduced the expression of both total *p73 *(Additional File 3 Figure S3A, lower panel) and *TA-p73 *(not shown) in the parental cells. Strikingly, basal p73 expression was strongly reduced upon transformation. However, transformed VH10 and HER cells showed comparable E2F1 regulation and *p73 *expression, so this cannot explain the observed differences in Nutlin-3 sensitivity. Therefore, these dissimilarities are more likely the result of different HA-hMDMX levels.

hMDMX overexpression in VH10 cells also inhibited p53 activation by 5-FU and etoposide on both mRNA and protein level (Additional File 3 Figure S3B and S3C), similar as observed with Nutlin-3 treatments. However, reduced proliferation in response to these drugs occurred mainly through p53-independent pathways, because neither p53-knockdown nor hMDMX overexpression did not rescue the growth inhibitory effect (Additional File 3 Figure S3D). Nevertheless, FACS analysis showed different responses between wild-type, hMDMX-overexpressing and p53-knockdown VH10 cells (data not shown). Upon etoposide treatment, wild-type and hMDMX-overexpressing cells showed a two-fold reduction of G1 phase and an increased G2 fraction. The G2 arrest in p53-knockdown cells was much more severe, with less than 10% remaining in G1. 5-FU induced S-phase accumulation and G2 reduction in wild-type and HA-hMDMX expressing cells, whereas p53-knockdown cells strongly accumulated in G1.

## Discussion

In this study, we analyzed the putative oncogenic function of hMDMX in the neoplastic transformation of normal human skin fibroblasts (VH10) and Human Embryonic Retinoblasts (HER). We chose retinoblasts since hMDMX is frequently overexpressed and/or amplified in retinoblastoma development. Retinoblastomas, like most other human tumors with increased hMDMX levels, retain wild-type p53 [19-22], suggesting that the oncogenic function of hMDMX is primarily based upon p53 inhibition. After RB inactivation, E2F1 is activated resulting in elevated p14ARF levels, repression of hMDM2 and activation of p53. Since hMDMX is not inhibited by p14ARF, hMDMX-overexpressing cells escape the p53-mediated cell death [38].

Indeed, we find that constitutive expression of hMDMX in foreskin fibroblasts functionally strongly resembles p53-knockdown cells. In combination with other defined genetic changes, hMDMX expression contributes to neoplastic transformation. In transformed cells, hMDMX overexpression reduces basal mRNA and protein levels of p53 targets, with exception of hMDM2 protein levels which are increased most likely via hMDMX-mediated stabilization. *Vice versa*, the expression of p53-repressed genes, like *SURVIVIN*, is increased. The ultimately obtained transformed cells show anchorage-independent growth, and can form tumors in an *in vivo *model.

Similarly, hMDMX-expressing HER cells largely resemble p53-knockdown HER cells regarding transformed properties, although hMDMX is less able to counteract the oncogenic HRas-induced growth inhibition, even in RB-knockdown cells. The ultimately obtained transformed cells, with either hMDMX overexpression or p53-knockdown, show *in vivo *growth capacity, although limited.

Our results support the idea that the hMDMX overexpression, which is found in a subset of human tumors [19-22], is an important step in the development of that tumor, and that its main function is to inactivate p53. Interestingly, recently two transgenic mouse models have been described that widely overexpress MDMX [39,40]. Surprisingly, the phenotypes were very different. Whereas mice from the Lozano lab spontaneously developed tumors upon MDMX overexpression [39], no spontaneous tumor formation nor cooperation with Eμ-Myc-induced tumors was observed in the mice from the Marine lab [40]. In both cases the MDMX-overexpressing MEFs or thymocytes showed an attenuated p53 response upon Nutlin-3 and IR treatment, respectively, suggesting the expression of a functional MDMX protein. It will be important to carefully examine these two mouse models to understand the distinct phenotype. This might teach us more about functions of MDMX in tumorigenesis.

In line with these studies, we find that hMDMX overexpression attenuates the Nutlin-3 mediated p53 activation and growth inhibition in skin fibroblasts. Nutlin-3 has a much lower affinity for hMDMX compared to hMDM2 [22,41], so the effect of hMDMX overexpression is probably caused by direct p53 inhibition. Similarly, hMDMX overexpression reduces p53 activation by etoposide and 5-FU.

More strikingly, the hMDMX-overexpressing HER cells are still sensitive to Nutlin-3. The p53-response is hardly affected, both regarding regulation of p53 target genes and inhibition of cell proliferation. This difference with hMDMX-transformed VH10 cells is probably due to the lower hMDMX levels in HER-hMDMX cells, which are even further reduced by Nutlin-3. In that respect, the Nutlin-3 response of the transformed retinoblasts resembles that of retinoblastoma cell lines. As we have shown before, these retinoblastoma cell lines are still sensitive to Nutlin-3 and even show an apoptotic response, despite high levels of hMDMX [22].

High hMDMX expression has been reported to attenuate the Nutlin-3 response [42-44]. In a study by Patton and colleagues [43], human embryonic lung fibroblasts were transformed using hTERT, E1A, and oncogenic Ras, with either hMDMX or hMDM2 overexpression, or p53-knockdown, and Nutlin-3 sensitivity was assessed. They found that hMDMX overexpression, in contrast to hMDM2, prevented p53 activation upon Nutlin-3 treatment, which fits most of our data. Nutlin-3 did not inhibit soft agar growth of hMDMX-overexpressing cells in their study. By contrast, we found partial inhibition of soft agar growth by Nutlin-3, whereas growth in a monolayer was not affected at all. Possibly, in 3D additional stress is posed upon the cells, causing super-activation of p53 that cannot be completely counteracted by hMDMX proteins.

The discussed fibroblast models also show a different Nutlin-3 response: IMR-90 cells entered apoptosis, whereas in VH10 cells Nutlin-3 mainly inhibited cell growth without induction of apoptosis (data not shown). Notably, IMR-90 cells are embryonic lung cells; embryonic cells are less differentiated and can be more easily transformed. Furthermore, Patton *et al*. used adenovirus E1A for RB inactivation, but E1A proteins have additional growth affecting functions, including attenuation of the p53 response by interacting with p300/CBP [45,46]. Therefore, a clean appreciation of the effects of hMDMX on the p53 response in the presence of E1A is difficult.

Beside hMDMX levels, also other factors may be involved in determining the outcome of Nutlin-3 treatment. Kitagawa *et al*. [36] have shown that RB status and E2F1 activity are important contributors. However, we found only minor changes in E2F1 activity in our model, which cannot explain the differences in Nutlin-3 sensitivity. Interestingly, the E2F1 target *TA-p73 *was dramatically decreased upon transformation. This might be a result of HRasV12 activity; oncogenic Ras has been described to switch the expression from TA-p73 to the antagonistic ΔN-p73, an important step during transformation. TA-p73 was reported to prevent anchorage-independent growth via activation of KCNK1 [47]. However, since transformed fibroblasts as well as retinoblasts express low levels of p73, this does not provide an explanation for the differential Nutlin-3 responses.

## Conclusions

In conclusion, we find that hMDMX overexpression can replace loss of p53 during the transformation process of human fibroblasts and embryonic retinoblasts. In addition, very high hMDMX levels, as observed in VH10 cells, can prevent p53 activation by Nutlin-3. However, lower hMDMX levels like in the HER cells can no longer inhibit p53 after Nutlin-3 treatment, because hMDMX protein is mostly degraded by elevated hMDM2 levels, as previously shown in other tumor cells [48]. The Nutlin-3 response of the transformed HER cells resembles that of retinoblastoma cell lines, indicating that this is a physiologically relevant model. A combination therapy using Nutlin-3 and a specific hMDMX inhibitor, possibly a low dose of a DNA damaging agent leading to hMDMX degradation, might result in more effective treatment of tumors expressing wild-type p53 and high levels of hMDMX.

## Methods

### Generation of stably transformed human cell lines

Primary human fibroblasts (VH10) and Human Embryonic Retinoblasts (HER) were immortalized by introducing human Telomerase (hTERT). Cells were maintained in DMEM supplemented with 10% FBS, 1% glutamine, antibiotics, amino-acids, glucose and vitamins. Stably transformed cell lines were generated in subsequent retroviral infection rounds according to the transformation schemes in Figure 1A and 1C. pRetroSuper-shRB-Hygro and pRS-Hygro [25] were used for RB-knockdown or control cell lines, followed by hygromycin selection (50 μg/ml). pMSCV-blast-Ras or pMSCV-blast [25] were used for HRasV12 overexpression or control cell lines, followed by blasticidin selection (5 μg/ml). pBABE-HA-hMDMX-puro or pRS-shp53-puro [25] were used for hMDMX overexpression or p53-knockdown, both combined with pMSCV-GFP-st [25] for SV40-small-t expression, followed by puromycin selection (0.5 μg/ml). Cell lines were maintained under selection pressure.

### Immunoblotting

Cells were lysed in Giordano 250 buffer (50 mM Tris-HCl, pH 7.4, 250 mM NaCl, 0.1% Triton X-100, 5 mM EDTA), with protease- and phosphatase inhibitors. Proteins were separated by SDS-PAGE, transferred onto polyvinyldene difluoride membranes (Immobilon-P, Millipore) and incubated with the appropriate primary (listed in Table 4) and HRP-conjugated secondary antibody (Jackson Laboratories). Bands were visualized by enhanced chemiluminescence (Super Signal; Pierce). Alternatively, membranes were incubated with secondary antibodies coupled to IRdye-680 and IRdye-800 near Infrared dyes (LI-COR Biosciences), and analyzed with the Odyssey Infrared Imager (LI-COR Biosciences). Signals were quantified using the Odyssey 2.1 analysis software (LI-COR Biosciences).

**Table 4:** List of used antibodies

Protein	Name/cat. #	Company
hMDMX	A300-287A	Bethyl Laboratories, Montgomery TX, USA

hMDMX	6B1A	[50]

HA-tag	HA.11	Covance, Princeton, New Jersey, USA

HA-tag	ab9110	Abcam, Cambridge, UK

p53°	DO-1/sc-126	Santa Cruz Biotechnology, Santa Cruz, CA, USA

p53°	PAb 1801/sc-98	Santa Cruz Biotechnology, Santa Cruz, CA, USA

p53	FL-393	Santa Cruz Biotechnology, Santa Cruz, CA, USA

hMDM2 */MDM2	4B2	[51]

hMDM 2 *	SMP14 sc-6965	Santa Cruz Biotechnology, Santa Cruz, CA, USA

p21	CP74/05-655	Upstate Biotechnology, Lake Placid, NY, USA

RB	G3-245/554136	BD Pharmingen, Franklin Lakes, New Jersey, USA

HRas	Y13-259	[52]

HAUSP/USP7	A300-033A	Bethyl Laboratories, Montgomery TX, USA

HAUSP/USP7	7G9	[53]

p21	CP74/05-655	Upstate Biotechnology, Lake Placid, NY, USA

γ-tubulin	GTU-88/T6557	Sigma-Aldrich, St Louis, MO, USA

Antibodies used in this study are listed in table 4, together with name or category number and the name of the company. For detection of human p53 we used a mix of DO-1 and 1801 (°), for detection of human Hdm2 we used a mix of 4B2 and SMP14 (*).

### Immunofluorescence

Cells were fixed with 4% paraformaldehyde for 10 min, permeabilized with 0.2% Triton X-100 for 10 min, blocked with 5% Normal Goat Serum (NGS) for 1 hour and incubated with primary antibodies for 1.5 hours and anti-mouse-Rhodamine secondary antibody (Jackson Laboratories) for 30 min. Coverslips were mounted onto microscope slides using DAPI-DABCO mounting solution.

### RNA isolation, qRT-PCR

RNA was isolated using the SV Total RNA isolation kit (Promega, Madison, WI). cDNA was synthesized using 1.0 μg RNA in Reverse Transcriptase reaction mixture (Promega). Samples were analyzed in triplicate using SYBR Green mix (Roche Biochemicals, Indianapolis, IN) in a 7900 ht Fast Real-Time PCR System (Applied Biosystems, Foster City, CA). For normalization the geometric mean of at least two housekeeping genes was used. Primer sequences are available in Table 5.

**Table 5:** Primer sequences

hMDM2-P2 Fw	5'-acgcacgccactttttctct- 3'
hMDM2-P2 Rv	5'-tccgaagctggaatctgtgag- 3'
P53 Fw	5'-ctctccccagccaaagaagaa- 3'
P53 Rv	5'-tccaaggcctcattcagctct- 3'
hMDMX Fw	5'-aggtgcgcaaggtgaaatgt- 3'
hMDMX Rv	5'-ccatatgctgctcctgctgat- 3'
PUMA Fw	5'-gacctcaacgcacagta- 3'
PUMA Rv	5'-ctaattgggctccatct- 3'
p21 Fw	5'-agcagaggaagaccatgtgga- 3'
p21 Rv	5'-aatctgtcatgctggtctgcc- 3'
SURVIVIN Fw	5'-gagacagaatagagtgatagg- 3'
SURVIVIN Rv	5'-gacagatgtgaaggttgg- 3'
GADD45A Fw	5'-gcgacctgcagtttgcaata- 3'
GADD45A Rv	5'-atcccccaccttatccatcct- 3'
CAPNS1 Fw	5'-atggttttggcattgacacatg- 3'
CAPNS1 Rv	5'-gcttgcctgtggtgtcgc- 3'
TBP Fw	5'-cacgaaccacggcactgatt- 3'
TBP Rv	5'-ttttcttgctgccagtctggac- 3'
RPS11 Fw	5'-aagcagccgaccatctttca- 3'
RPS11 Rv	5'-cgggagcttctccttgcc- 3'
SRPR Fw	5'-cattgcttttgcacgtaaccaa- 3'
SRPR Rv	5'-attgtcttgcatgcggcc- 3'
CDC25A Fw	5'-ctccgagtcaacagattcagg- 3'
CDC25A Rv	5'-ttcaaggttttctttactgtccaa- 3'

Sequences of qRT-PCR primers used in this study.

### Growth assay, soft agar assay

For growth assays, 1000 cells were seeded in triplicate in 96-wells plates; treatments were started 24 hours after seeding. Cells were incubated with WST-1 reagent (Roche) for 1-4 hours and absorbance (450 nm) was measured in a microplate reader (Victor3 Multilabel Counter 1420-042, Perkin-Elmer).

Soft agar assays were performed in 96-well plates (VH10) or 6-well plates (HER) coated with a 0.6% agarose bottom-layer. Per well, 5000 VH10 or 20.000 HER cells were seeded in 0.3% agarose. Colony outgrowth was monitored (10 × magnification, Olympus CKX41) and pictures were taken at several time points.

### Flow cytometry

Cells were harvested, washed with PBS and fixed ice-cold 70% ethanol. Cells were washed in PBS and incubated in PBS containing 50 μg/ml propidium iodide and 50 μg/ml RNase. Flow cytometry was performed in a BD LSR II system (BD Biosciences).

### Cytogenetic methods and combined binary ratio fluorescence in situ hybridization (COBRA-FISH)

Culturing, harvest conditions and karyotyping were performed according to standard protocols [49]. Slides with metaphase chromosomes were hybridized using a multicolor FISH approach. Staining, digital imaging, and analysis were performed as described previously [30]. Hybridizations with individual libraries labeled with single fluorochromes were used to confirm the detected rearrangements. Chromosomal breakpoints were assigned by using inverted images counterstained with 4',6-diamidino-2-phenylindole (DAPI; Downers Grove, IL) together with the information derived from the short- and long-arm specific hybridization during COBRA-FISH. Karyotypes were described according to ISCN 2009.

### Shell-less Chicken Chorioallantoic Membrane (CAM) assay

Fertilised chicken eggs were incubated at 37°C in humidified atmosphere. After 4 days they were cracked open into plastic dishes. At day 7, two-and-half million cells transduced with turbo-GFP lentiviral construct (SHC003, Sigma-Aldrich) were mixed with 50 μl basement membrane matrix (BD Biosciences) and grafted onto the CAM. At day 17, tumors with surrounding CAM were removed and the size was measured. GFP-positive tumors were photographed using a fluorescence stereomicroscope. Tumors were embedded in paraffin, sectioned and stained with anti-Vimentin, clone V9 (Santa Cruz) and anti-phospho-Histone-3 (Upstate, Millipore). Percentage of proliferating cells was calculated by quantifying phospho-Histone-3 positive nuclei of on average 500 nuclei from 5 random pictures per sample.

## Competing interests

The authors declare that they have no competing interests.

## Authors' contributions

KLe and JdL performed the transformations of the human cells, performed all growth assays and contributed to the protein and mRNA analyses and immunofluorescence data. KLo and EW performed the *in vivo *tumorigenicity studies. AT, KLo and MVdV performed protein and mRNA analyses and contributed to the immunofluorescence data. MvdB and KS performed and interpreted the COBRA-FISH analyses. KLe, JdL and AGJ designed and coordinated the study and drafted the manuscript. All authors have read and approved the final manuscript.

## Supplementary Material

Additional file 1 **Figure S1**. Overexpressed HA-hMDMX is localised both nuclear and cytoplasmic and does not alter p53 and hMDM2 localisation. Localisation of hMDMX, hMDM2 and p53 in various VH10 (A, B) and HER (C, D) cell lines was determined by immunofluorescence using the indicated antibodies. DAPI staining was used to visualise nuclei, GFP signal represents SV-40 small-t expression. Click here for file

Additional file 2 **Figure S2**. Karyotyping of VH10 and HER cell lines. Karyotypes of transformed VH10 (A) and HER (B) cell lines using combined binary ratio labeling-fluorescence in situ hybridization (COBRA-FISH). Representative karyograms after COBRA-FISH hybridization are shown for each cell line. Click here for file

Additional file 3 **Figure S3**. hMDMX overexpression inhibits p53 response but does not rescue the growth inhibition induced by 5-fluoro-uracil or etoposide in human fibroblasts. A. The indicated VH10 and HER cell lines were treated with 10 μM Nutlin-3 for 24 hours and analyzed with qRT-PCR. Expression levels of *E2F1, CDC25a *(upper panel) and *p73 *(lower panel) were normalized for housekeeping genes *RPS11 *and *CAPNS1*. B. Indicated VH10 cell lines were treated for 24 hours with 25 μM 5-FU, 5 μM etoposide or mock treated, and analyzed with qRT-PCR. Expression levels of *hMDM2-p2 *and *p21 *were normalized for housekeeping genes *CAPNS1 *and *SRPR*. C. Protein levels of cells treated as in B were analyzed with immunoblotting using the indicated antibodies. D. Cell growth was monitored using WST-1 proliferation assays. 24 hours after seeding the cells were treated for 24 hours with the indicated drugs. Cell proliferation was measured at day 0, 2 and 4 after treatment. Click here for file
