# Supplementary figures and images for: Oncogenic functions of hMDMX in in vitro transformation of primary human fibroblasts and embryonic retinoblasts
Source: Mol Cancer. 2011 Sep 12;10:111. doi: 10.1186/1476-4598-10-111 (PMC3179748; doi:10.1186/1476-4598-10-111)

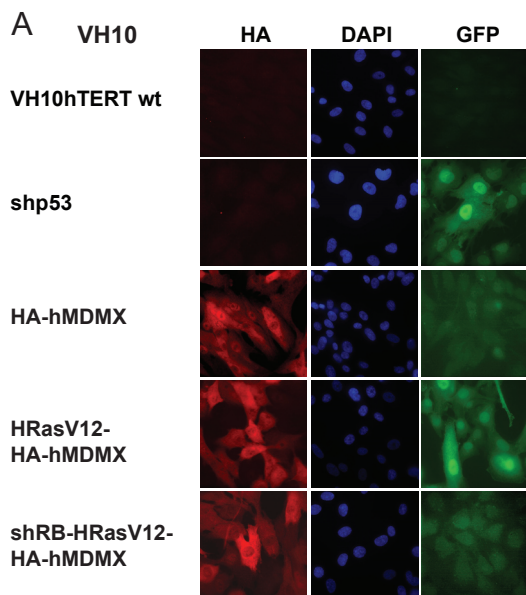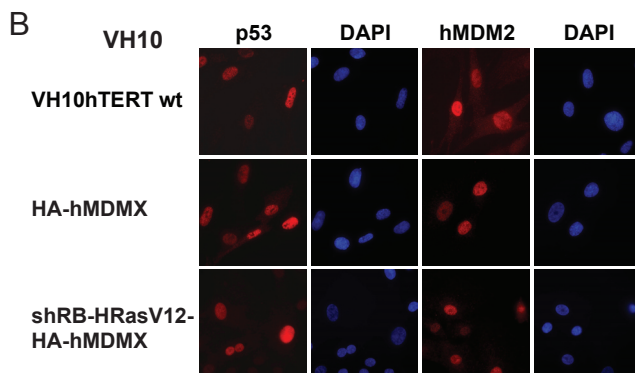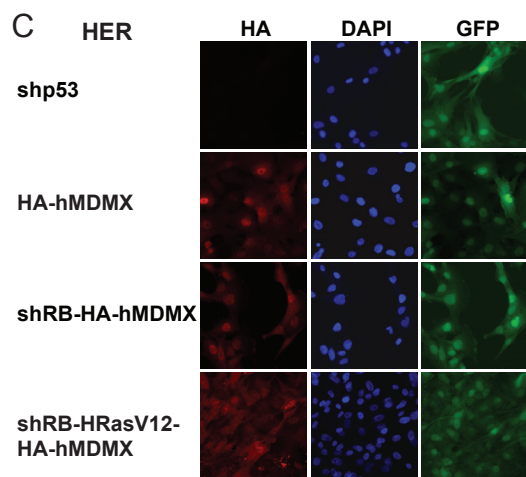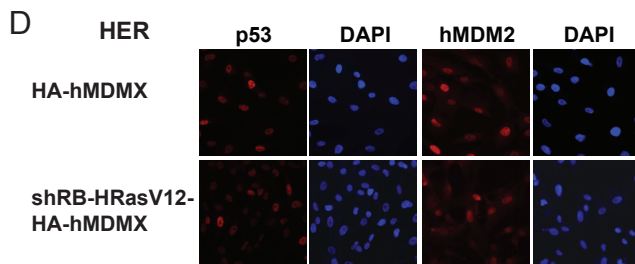

Supplement: Additional file 1 — Figure S1. Overexpressed HA-hMDMX is localised both nuclear and cytoplasmic and does not alter p53 and hMDM2 localisation. Localisation of hMDMX, hMDM2 and p53 in various VH10 (A, B) and HER (C, D) cell lines was determined by immunofluorescence using the indicated antibodies. DAPI staining was used to visualise nuclei, GFP signal represents SV-40 small-t expression. [file 1476-4598-10-111-S1.PDF]

A

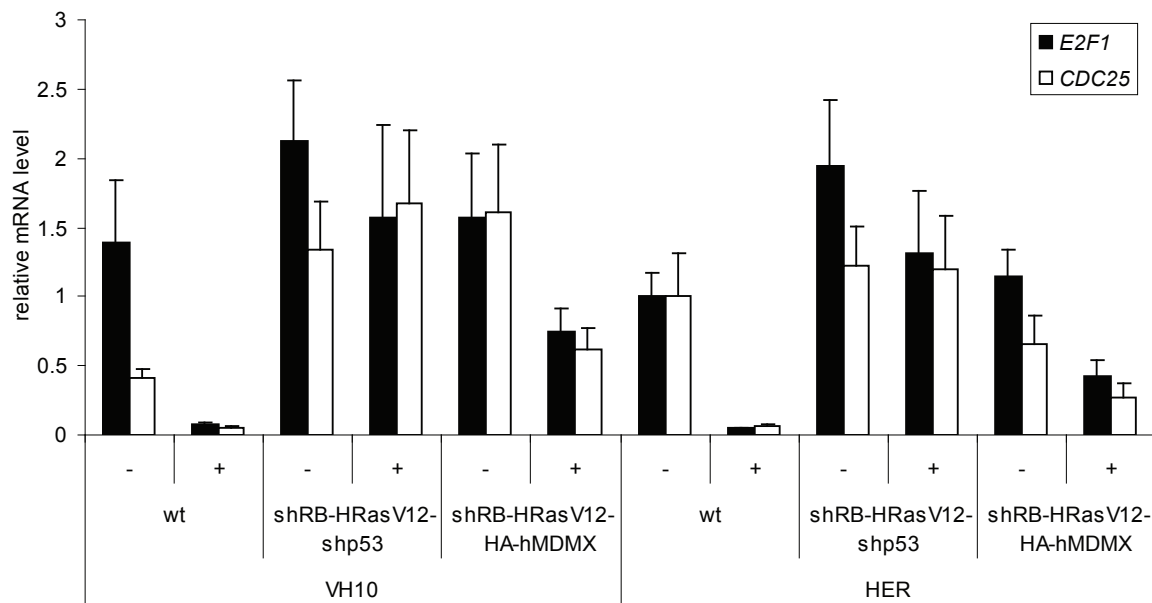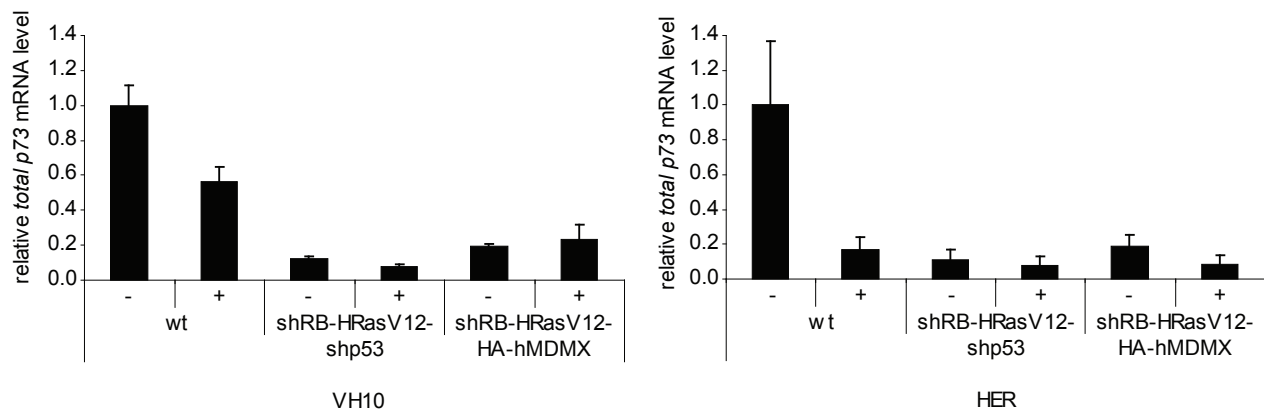

B

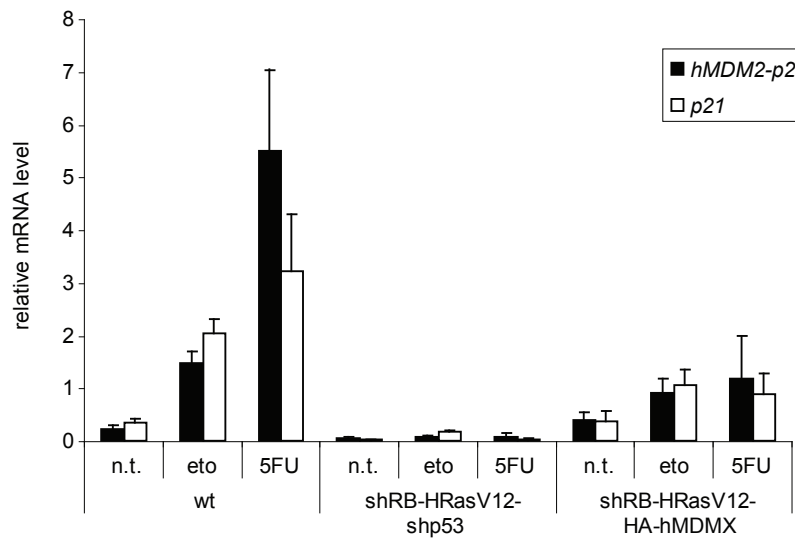

C

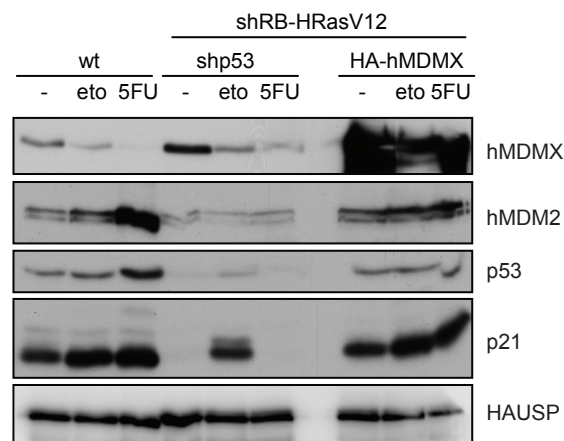

D

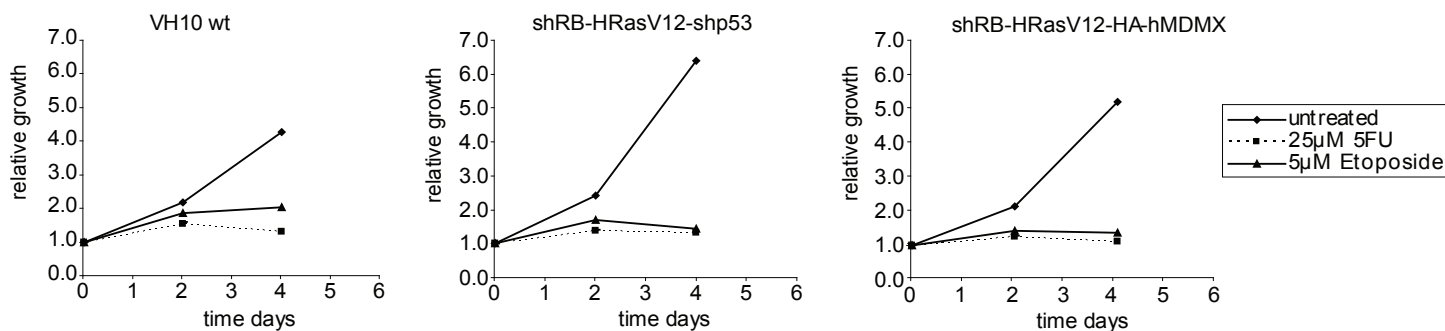

Supplement: Additional file 3 — Figure S3. hMDMX overexpression inhibits p53 response but does not rescue the growth inhibition induced by 5-fluoro-uracil or etoposide in human fibroblasts. A. The indicated VH10 and HER cell lines were treated with 10 μM Nutlin-3 for 24 hours and analyzed with qRT-PCR. Expression levels of E2F1, CDC25a (upper panel) and p73 (lower panel) were normalized for housekeeping genes RPS11 and CAPNS1. B. Indicated VH10 cell lines were treated for 24 hours with 25 μM 5-FU, 5 μM etoposide or mock treated, and analyzed with qRT-PCR. Expression levels of hMDM2-p2 and p21 were normalized for housekeeping genes CAPNS1 and SRPR. C. Protein levels of cells treated as in B were analyzed with immunoblotting using the indicated antibodies. D. Cell growth was monitored using WST-1 proliferation assays. 24 hours after seeding the cells were treated for 24 hours with the indicated drugs. Cell proliferation was measured at day 0, 2 and 4 after treatment. [file 1476-4598-10-111-S3.PDF]
