# Supplementary material for: Oncogenic functions of hMDMX in in vitro transformation of primary human fibroblasts and embryonic retinoblasts
Source: Mol Cancer. 2011 Sep 12;10:111. doi: 10.1186/1476-4598-10-111 (PMC3179748; doi:10.1186/1476-4598-10-111)
Supplement: Additional file 2 — Figure S2. Karyotyping of VH10 and HER cell lines. Karyotypes of transformed VH10 (A) and HER (B) cell lines using combined binary ratio labeling-fluorescence in situ hybridization (COBRA-FISH). Representative karyograms after COBRA-FISH hybridization are shown for each cell line. [file 1476-4598-10-111-S2.PDF]

A

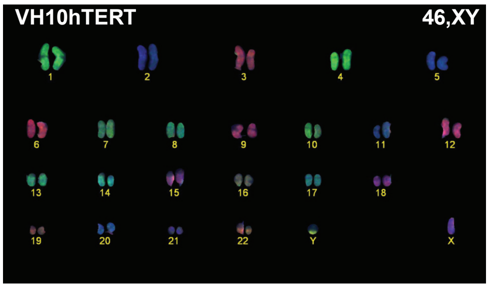

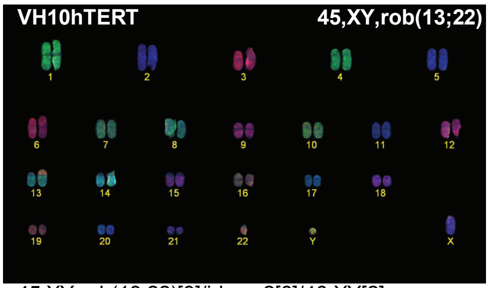

45,XY,rob(13;22)[8]/idemx2[3]/46,XY[2]

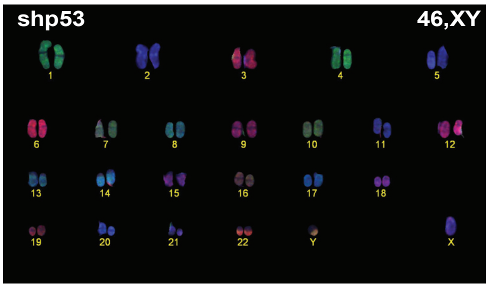

46,XY[4]/idemx2[3]/45,XY,rob(13;22)[2]/idemx2[1] Additional, random translocation in some cells.

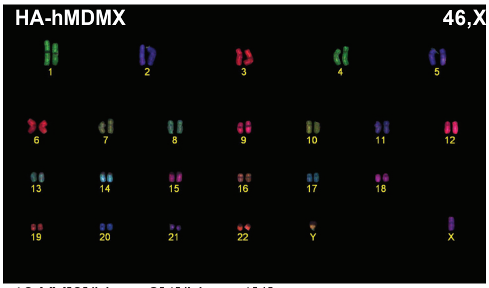

46,XY[8]/idemx2[4]/idemx4[1]

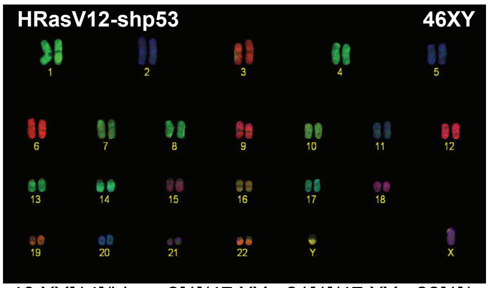

46,XY[14]/idemx2[1]/47,XY,+21[1]/47,XY,+22[1]/91,XXYY,t(1;16)[1]/90,XXYY,t(4;16),t(16;21),-9[1]

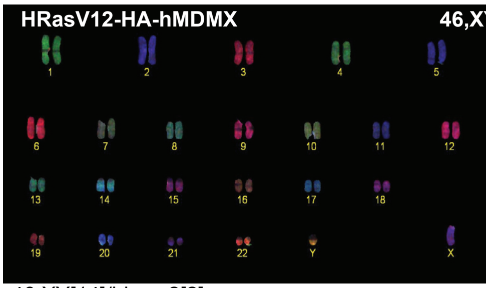

46,XY[14]/idemx2[2]

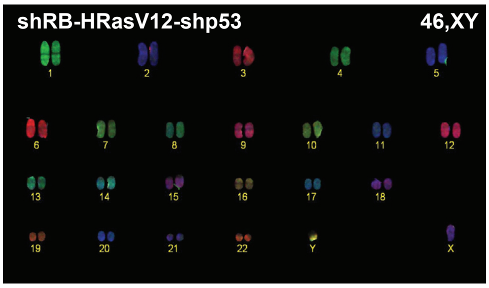

46,XY[17]/idemx2[1]/47,XY,-6,+8,+19[1]

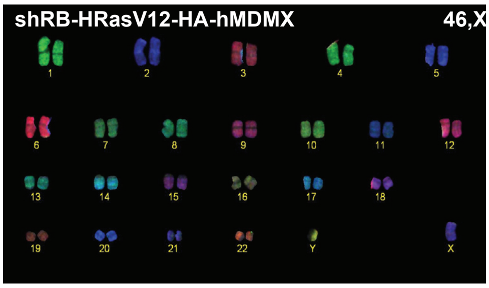

46,XY[15]/idemx2[1]

B

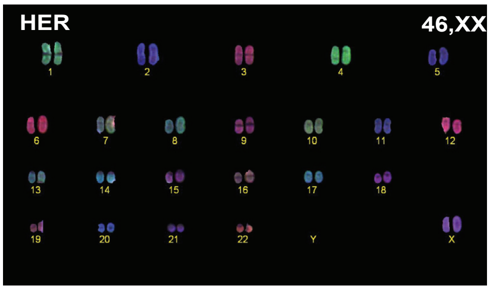

46,XX/idemx2

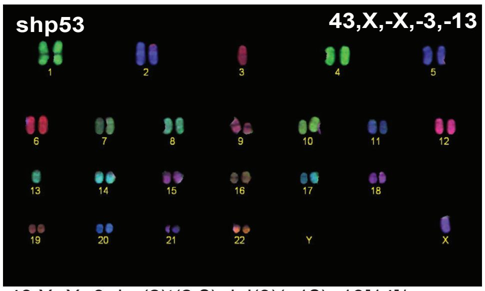

43,X,-X,-3,der(2)t(2;?),del(9)(p1?),-13[14]/idemx2[4],idemx4[2] Additional, non-clonal changes were seen in 5 metaphases.

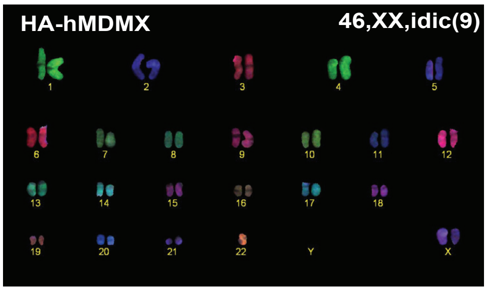

46,XX,idic(9)(p1?)[19]/idemx2[1]

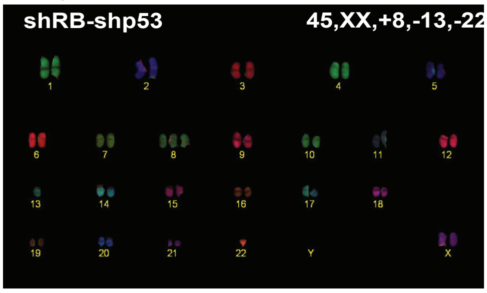

45,XX,+8,-13,-22[10]/idemx2[5]

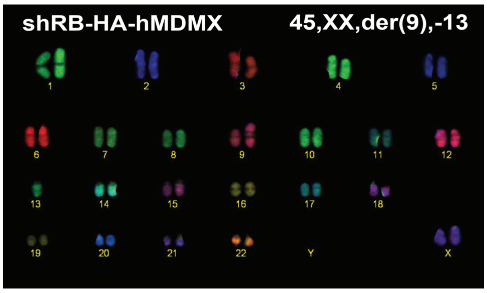

45,XX,-13,der(9)t(9;9)(p1?;q10)[17]/idemx2[3]

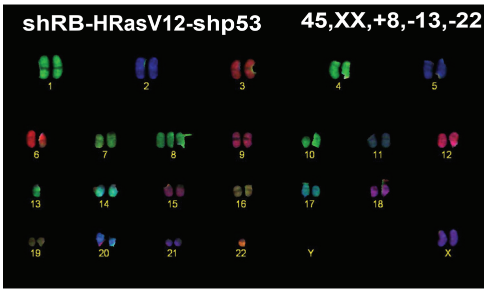

45,XX,der(6)t(6;19)+8,-13,-22[3]/idemx2[4]44,XX,+ider(8)t(5;8)(q?;q?),-13,-22[3]/idemx2[6]

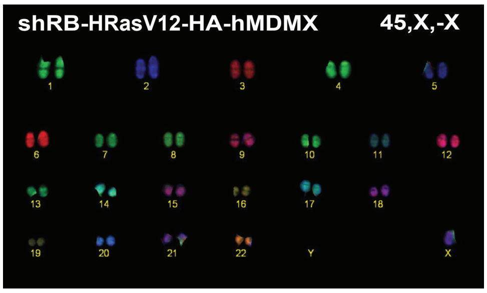

45,X,-X[16]/idemx2[2]45,X,-X,der(13;19)(q;q),-19[2] Some cells with random centric fusion or telomeric associations
